# Pineapple consumption reduced cardiac oxidative stress and inflammation in high cholesterol diet-fed rats

**DOI:** 10.1186/s12986-021-00566-z

**Published:** 2021-04-07

**Authors:** Porrnthanate Seenak, Sarawut Kumphune, Wachirawadee Malakul, Ratanon Chotima, Nitirut Nernpermpisooth

**Affiliations:** 1 Integrative Biomedical Research Unit (IBRU), Faculty of Allied Health Sciences, Naresuan University, Phitsanulok, 65000 Thailand; 2 Department of Cardio-Thoracic Technology, Faculty of Allied Health Sciences, Naresuan University, Phitsanulok, 65000 Thailand; 3 Biomedical Engineering Institute (BMEI), Chiang Mai University, Chiang Mai, 50200 Thailand; 4 Department of Physiology, Faculty of Medical Sciences, Naresuan University, Phitsanulok, 65000 Thailand; 5 Department of Chemistry, Faculty of Sciences, Naresuan University, Phitsanulok, 65000 Thailand

**Keywords:** Pineapple, Oxidative stress, Cardiac inflammation, Hypercholesterolemia

## Abstract

**Background:**

Hypercholesterolemia is a major risk factor for cardiovascular disease. It has been reported that pineapple contains healthy nutrients and phytochemicals associated with antioxidant and anti-inflammatory capacities. No investigation exists concerning the effect of pineapple consumption modulating hypercholesterolemia-induced cardiac damage in high-cholesterol diet (HCD)-fed rats. This study evaluated the effect of pineapple consumption on lipid-lowering, cardiac oxidative stress and inflammation in HCD-fed rats.

**Methods:**

Male Sprague–Dawley rats were fed with HCD, in the presence and absence of Pineapple (*Ananas comosus* L.) cv. Pattavia powder for 8 weeks. Then, serum lipid profiles, liver and renal function tests, cardiac oxidative stress and pro-inflammatory cytokines were determined.

**Results:**

Daily pineapple consumption reduced weight gain, serum lipid profiles, atherogenic coefficient (AC), cardiac risk ratio (CRR), and liver enzyme activity, without causing renal dysfunction. Pineapple consumption also restores cardiac protein carbonyl (cPC) content, reduces cardiac malondialdehyde (MDA), cardiac pro-inflammation cytokine IL-6 and IL-1β levels.

**Conclusion:**

Pineapple possesses antioxidant and lipid-lowering properties and daily consumption alleviates hypercholesterolemia-induced cardiac lipid peroxidation and pro-inflammation elevation in an in vivo model. This study demonstrates that pineapple is a potential candidate for cardioprotection against hypercholesterolemia.

## Introduction

Hypercholesterolemia is a major metabolic disorder, which is considered a major risk for cardiovascular morbidity and mortality. Outcomes of unhealthy high-cholesterol diet (HCD) consumption or familial hypercholesterolemia (FH) abnormally increase lipids in the circulation and accumulation in particular tissues [1]. Lipids are the main source of energy required for cardiac pumping function [2–4]. Prolonged HCD-induced hypercholesterolemia has been implicated in lipid accumulation in the heart [4–7]. Excessive lipid overload in the myocardium is prone to be harmful by disrupting ion channels and membrane integrity [2–8]. Lipid oversupply into the heart and fat accumulation in cardiomyocytes, produces lipotoxicity from fatty acid oxidation and uncoupling mitochondrial ROS production, which leads to myocyte loss through apoptosis [2–5, 9, 10]. These highly reactive species mediate low-grade inflammation by inducing oxidative damage locally to cardiac lipid and protein molecules [10, 11]. Therefore, the prevention of HCD-induced myocardial inflammation could be a feasible approach to prevent deleterious cardiac complications.

Nowadays, lipid-lowering therapies are not only targeting lipid profiles to lower the risk of atherosclerotic cardiovascular disease (ASCVD), but also focusing on cholesterol-independent mechanisms such as cardiac endpoints [12, 13]. For instance, statin is a first-line drug for treating dyslipidaemia with additional pleiotropic effects on both antioxidation and anti-hypertrophy which are associated with the improvement of the heart structure and its functions [14]. Despite the widespread usage of this drug, long-term treatment should be investigated regarding its potential complications and non-adherence that may cause major clinical implications in certain patients [12]. Alternatively, eating functional foods that are healthy and contain hypolipidemic and antioxidant properties could be an attractive choice for helping to manage hyperlipidaemia [15].

Pineapples are a popular tropical fruit that have a high nutritional value and medicinal properties. They are consumed worldwide and are easily incorporated into daily diets. It contains an abundance of dietary fibre and phytochemicals such as gallic acid, catechin, epicatechin and ferulic acid. These biologically active compounds provide powerful natural pharmacological activities [16, 17]. Several studies have reported on the health benefits of pineapple consumption as highlighted by anti-obesity, anti-dyslipidaemia, antioxidant, and anti-inflammatory properties [16–19]. However, few studies have focused on the effect of pineapple consumption on cardiac oxidative stress and inflammation in hypercholesterolemia model. Here, we hypothesized that daily pineapple intake could reduce cardiac oxidative stress and inflammation in HCD-induced hypercholesterolemia rats. This study will provide information for the cardioprotective effect of pineapple consumption in relation to dyslipidaemia.

## Materials and methods

### Pineapple preparation

Pineapple (*Ananas comosus* L.) cv. Pattavia which is grown in Thailand was used in this study. The pineapples were weighed, sliced, and dried at 60 °C for 96 h using a hot air oven. The dried pineapple slices were weighed and ground into fine powder, then stored at -20 °C until further study.

### Evaluation of active compounds, antioxidant capacity and approximate analysis

Total phenolic content was determined by using Folin–Ciocalteu’s colorimetric method [20]. 10 mg of dried pineapple was incubated in 1 mL ethanol for 48 h and then sonicated for 20 min. The samples were filtered, then 20 µL of the solution was transferred into a 96-well microplate and mixed with 100 µL of Folin–Ciocalteu’s working reagent. After 5 min 80 µL of 7.5% (w/v) Na_2_CO_3_ was added and incubated in a dark room for 30 min. The absorbance was measured at 765 nm and the total phenolic content was calculated by using the linearity of gallic acid.

Total flavonoid content was determined using the method described by Chang et al. [21]. 10 mg of dried pineapple was incubated with 1 mL ethanol for 48 h followed by sonication for 20 min. The samples were filtered, then 500 µL of the solution was mixed with 1.5 mL of ethanol. After that, 100 μL of 10% (w/v) Aluminum chloride (AlCl_3_) and 100 μL of 1 M Potassium acetate (CH_3_COOK) were added. The volume was made up to 5 mL using distilled water and placed in the dark at room temperature for 30 min. Quercetin was used as a standard curve at a wavelength 415 nm.

The 1,1-diphenyl-2-picrylhydrazyl (DPPH) assay described by Brand-Williams et al. [22] was performed to evaluate antioxidant capacity of the pineapple in this study. Dried pineapple was mixed with ethanol at the concentrations of 375, 750, 1,500, 3,000, 6,000, and 12,000 µg/mL, then 100 μL of the samples were transferred into a 96-well microplate and mixed with 100 μL of 200 μM DPPH followed by incubation in the dark for 30 min. The absorbance was measures at 517 nm and the antioxidant capacity was determined by using the linearity of the standard Trolox curve.

The 2,2′-azinobis-(3-ethylbenzothiazoline-6-sulfonate) (ABTS) method was described by Arnao et al. [23] to determine the pineapples antioxidant capacity. The dried pineapple was prepared with similar concentrations as above by using 50 μL of the samples mixed with 100 μL of working ABTS solution and then incubated for 15 min, followed by the Trolox being used as a standard curve at a wavelength of 734 nm.

The proximate components of the dried pineapple, which included ash, carbohydrates, fat, moisture, protein, and dietary fibre were analyzed at the Central Lab Thai, Bangkok, Thailand, according to standard protocols.

### Animal model

All animal experiments were approved by the Animal Ethics Committee of Naresuan University, Thailand (approval number: NU-AE610409), and performed according to the regulations of the institutional guidelines for the care and use of laboratory animals. Four week old Four-week-old male Sprague–Dawley rats that weighed 150–200 g were obtained from Nomura Siam International Co., Ltd. Bangkok, Thailand, and housed at the Centre for Animal Research, Naresuan University in Phitsanulok, Thailand. The rats were maintained in a controlled temperature environment at 25 ± 2 °C, a 12 h light‑dark cycle with a standard diet and filtered water ad libitum for 1 week. After the rats were acclimatized, they were randomly divided into 5 groups (n = 5–6) and treated for 8 weeks: control with standard diet and normal drinking water (control), high-cholesterol diet (standard diet + 1.5% (w/w) of cholesterol, 0.37% (w/w) of cholic acid) (HCD), high-cholesterol diet with a low-dose of pineapple; LPA (100 mg/kg/day) (HCD+LPA), high-cholesterol diet with a high-dose of pineapple; HPA (200 mg/kg/day) (HCD+HPA), and high-cholesterol diet with simvastatin (40 mg/kg/day) (HCD+S). Their body weights were determined weekly, and their total food consumptions were measured daily. The powdered pineapple and the simvastatin were administered by oral gavage.

### Heart and serum collection

At the end of the 8-weeks dietary schedule, the rats were fasted overnight then anesthetized with pentobarbital sodium (Nembutal® Sodium Solution CII; 100 mg/kg; Akorn, Inc., Lake Forest, IL, USA) and lithium heparin (150 U; Government Pharmaceutical Organization, Bangkok, Thailand) via an intraperitoneal injection. Then an operation on the rats was carried out to expose their thoracic cavities, after the incision area had been cleaned with70% (v/v) ethanol. Their hearts were weighed then snap-frozen in liquid nitrogen and stored at -80** °C**. The blood samples were collected rapidly from the thoracic cavity and centrifuged at 3000×*g* for 10 min at 4 °C. The Serum was then collected and immediately snap-frozen in liquid nitrogen and kept at − 20 °C, followed by its biochemical parameters being measured within 24 h.

### Determination of blood biochemistry

Total cholesterol (TC), high-density lipoprotein cholesterol (HDL-C), Low-density lipoprotein cholesterol (LDL-C), triglyceride (TG), aspartate amino-transferase (AST) activity, alanine amino-transferase (ALT) activity, blood urea nitrogen (BUN) as well as creatinine (Cr) were analyzed using an automate biochemistry analyzer (cobas c 111 analyzer). Atherogenic Coefficient (AC) was calculated by (TC-HDL-C)/HDL-C and Cardiac risk ratio (CRR) was evaluated following this formula; CRR = TC/HDL-C [24].

### Hearts homogenization and protein extraction

The frozen heart tissues were thawed and homogenized in cold phosphate buffer saline (PBS) containing a protease inhibitor cocktail (CAT. 0589295301, ROCHE, Germany; 100 µL/100 mg) by using a pestle and mortar. The homogenized tissues were then centrifuged at 14,000×*g* for 10 min at 4** °C** and the supernatants were collected for determining the protein concentration by using the Bradford assay (BIO-RAD, USA) as previously described [25].

### Protein carbonyl assay

To determine the protein carbonyls content in oxidized protein, the 2,4-dinitrophenylhydrazine (DNPH) spectrophotometric assay was performed as previously describe [26]. Pre-analytical quality controls that included linearity test, within-run precision assay, and between day precision were determined using the control serum (HUMATROL P) with the DNPH spectrophotometric assay.

### Determination of inflammatory cytokine levels

The determination of inflammatory cytokine levels that included tumor necrosis factor-alpha (TNF-α; CAT. 900-M73; Prepotech®, USA), interleukin 1-beta (IL1-β; CAT. 900-M91; Prepotech®, USA), and interleukin 6 (IL-6; CAT. 900-M86; Prepotech®, USA), were performed using the enzyme-linked immunosorbent assay (ELISA) Buffer Kit (CAT. 900-K00; Prepotech®, USA) according to the manufacturer’s instructions.

### Lipid peroxidation assay

Malondialdehyde (MDA) levels were measured by using the Lipid Peroxidation Assay Kit (ab118970; Abcam, Cambridge, UK). The hearts were homogenized on ice in 303 µL MDA lysis buffer and centrifuged at 14,000×*g* for 10 min at 4** °C**. This was followed by 200 µL of the supernatant being mixed with 600 µL of the thiobabituric acid (TBA) solution, incubated at 95 °C for 60 min and then cooled on ice for a further 10 min, after which 200 µL of the mixed solution was added to a 96-well microplate. The MDA level was calculated with standard curve at a wavelength 532 nm and calculated following the manufacturer's protocols.

#### Measurements of total antioxidant capacity of the heart tissue

The Total Antioxidant Capacity Assay Kit (ab65923; Abcam, Cambridge, UK) was used to evaluate the total antioxidant capacity (TAC) of the heart tissues. Then they were washed with cold phosphate buffer saline (PBS) and subsequently homogenized. The heart homogenate was incubated on ice for 10 min and centrifuged at 14,000×*g* for 10 min at 4** °C**. The supernatant was transferred to a 96-well microplate, then a 100 µL CU2+ of the working solution was added. After being incubated on a shaker for 90 min at room temperature in the dark, the absorbance was measures at 570 nm and the antioxidant capacities were determined by using the linearity of the standard Trolox curve according to the manufacturer's protocols.

#### Statistical analysis

All the data was expressed as the mean ± SEM. One-way analysis of variance (ANOVA) followed by Turkey’s post hoc analysis being performed using GraphPad Prism 5.0. *p* value < 0.05 was considered as statistically significance.

## Results

### Percentage yield, total phenolic content, total flavonoid content, and oxidant scavenging capacity of the pineapple

After drying 4500 g of fresh pineapple in a hot air oven, the total yield of powder was 600 g which equated to 13.33% of its original weight. The total phenolic and flavonoid contents were 11.17 mg GAE/100 g and 6.33 mg QE/100 g, respectively. To determine the antioxidant capacity of the pineapple, the DDPH assay and the ABTS oxidant scavenging method were performed. The pineapple had a dose-dependent effect on percentage oxidant scavenging. The maximal tested dose of dried pineapple at 12,000 µg/mL was 78.38 ± 0.91% by the DDPH method, while the ABST method was 97.93% (Table 1). The half maximal inhibitory concentration (IC_50_) values were calculated by Log[concentration] (data not shown). The IC_50_ of the pineapple on oxidant scavenging using the DDPH and ABTS methods were 5,619 μg/mL and 1,265 μg/mL, respectively. Proximate analysis showed the components of the dried pineapple had high carbohydrates at 87.72 g/100 g, low fat at 1.66 g/100 g and high dietary fiber at 9.07 g/100 g (Table 2). These results indicated that phenolic and flavonoid compounds were present in the pineapple powder with a high antioxidant capacity.

**Table 1:** Percentage oxidant scavenging of the dried pineapple

Concentration of pineapple (μg/mL)	% Oxidant scavenging (DPPH assay)	% Oxidant scavenging (ABTS assay)
375	5.65 ± 2.58	21.31
750	9.05 ± 2.90	34.35
1500	16.73 ± 2.35	50.18
3000	28.59 ± 3.02	64.22
6000	47.51 ± 3.09	90.16
12,000	78.38 ± 0.91	97.93

**Table 2:** The biochemical compositions of the dried pineapple

Analysis	Amount of analytes (g/100 g dry weight of pineapple)	Analysis methods
Ash	2.64	AOAC (2016) 920.153
Carbohydrate	87.72	In-house method TE-CH-169 based on Compendium of Methods for Food Analysis Thailand, 1st Edition, 2003
Fat	1.66	AOAC (2016) 922.06
Moisture	3.57	In-house method based on AOAC (2016) 934.06
Protein (%N × 6.25)	4.41	In-house method TE-CH-042 based on AOAC (2016) 981.10
Dietary Fiber	9.07	In-house method TE-CH-076 based on AOAC (2016) 985.29

### Effects of Pineapple consumption on body weight, heart weight/body weight ratio, blood parameters, atherogenic coefficient, and cardiac risk ratio

**Fig. 1:**
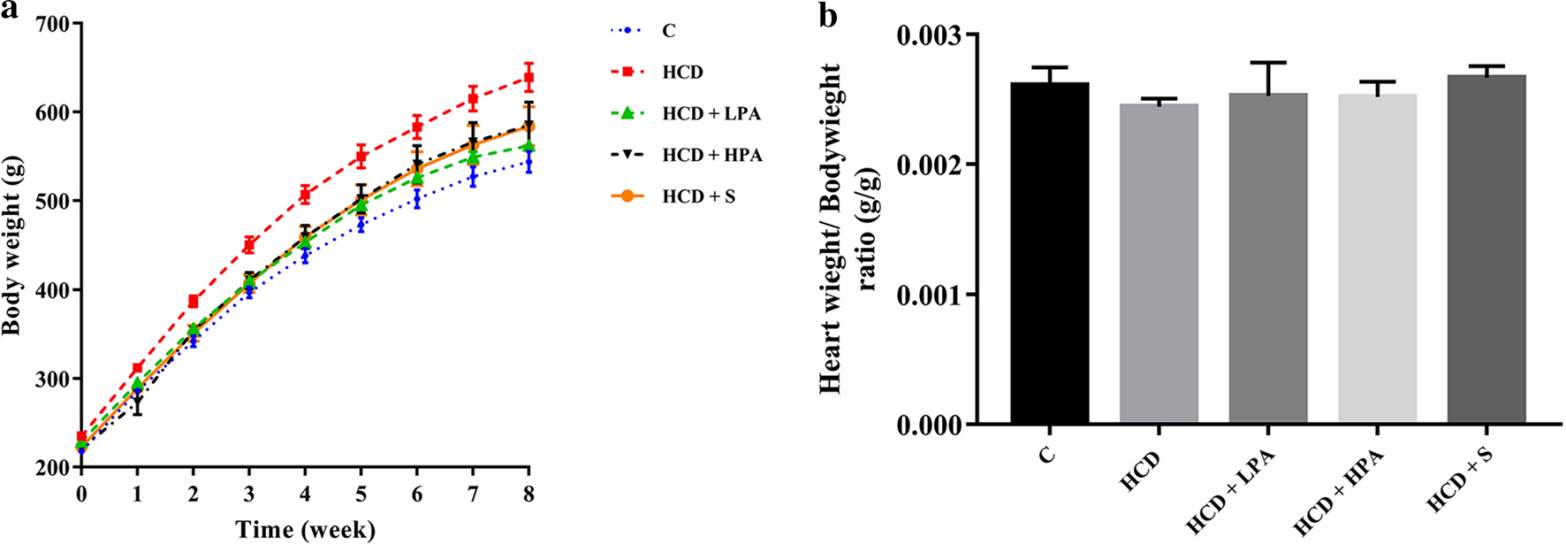
The determination of eight weeks of pineapple consumption on body weight (BW; g) and heart weight (HW; g) / BW ratio (g). **a** Representative growth curve of male Sprague–Dawley rats; **b** HW/BW ratio of experimental groups. Data were presented as mean ± SEM., (n = 5–6 per group)

**Fig. 2:**
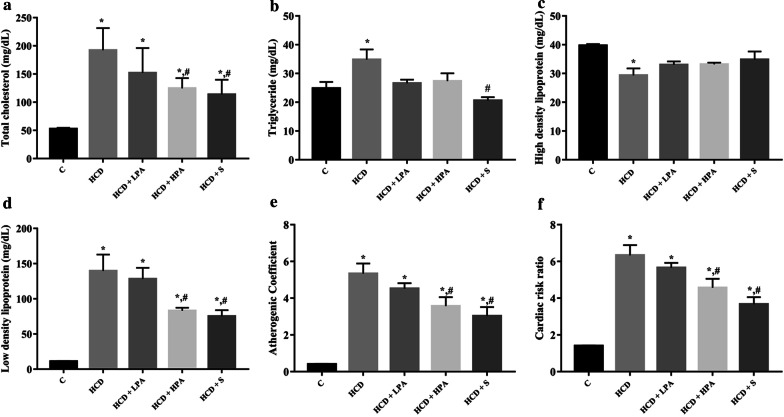
The effects of eight weeks of pineapple consumption on the serum lipid profiles in high-cholesterol fed rats. After 8-weeks, blood samples in all experimental groups were collected. Atherogenic Coefficient (AC) and Cardiac risk ratio (CRR) were calculated. **a** Total cholesterol (TC); **b** Triglyceride (TG); **c** High-density lipoprotein cholesterol (HDL-C); **d** Low-density lipoprotein cholesterol (LDL-C); **e** Atherogenic Coefficient (AC); **f** Cardiac risk ratio (CRR). Each bar presented in mean ± SEM. *p < 0.05 VS the control group, #*p* < 0.05 versus the HCD group, (n = 5–6 per group)

**Fig. 3:**
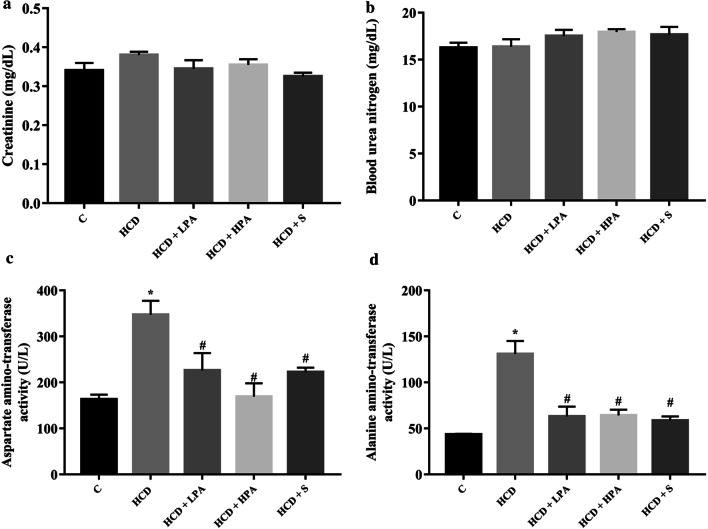
The effects of eight weeks of pineapple consumption on the renal and liver functions in high-cholesterol fed rats. **a** Creatinine (Cr); **b** Blood urea nitrogen (BUN); **c** Aspartate amino-transferase (AST) activity; **d** Alanine amino-transferase (ALT) activity. Each bar presented in mean ± SEM. *p < 0.05 VS the control group, #*p* < 0.05 versus the HCD group, (n = 5–6 per group)

Starting from the second week, the high cholesterol diet fed rats significantly increased their body weight when compared to the control group. Daily intake of 100 mg/kg of pineapple (LPA) or 200 mg/kg of pineapple (HPA) for 8 weeks significantly decreased body weight from the third week to the end of the experiment compared with the HCD group (*p* < 0.05) (Fig. 1a), while the food intake remained unchanged in all groups. At week 8, there were no significant differences in the heart and body weight ratios, Cr and BUN in all the groups (Figs. 1b, 3a, b). The administration of HPA dramatically reduced the TC, LDL-C, AST and ALT serum levels compared to the HCD group and similar changes were observed in the simvastatin treatment group (*p* < 0.05) (Figs. 2a, d, 3c, d). The TG serum showed a reducing trend in both the HCD+LPA and HCD+HPA groups (Fig. 2b). The HCD group showed significantly lower levels of HDL-C serum than the other groups, while there was a slight increase of HDL in both the HCD+LPA and HCD+HPA groups (Fig. 2c). The HCD-C group also had dramatically higher AC and CRR, while the HPA group showed significantly lower levels of them, which was similar to the simvastatin treated group (*p* < 0.05) (Fig. 2e, f).

These results indicated that daily consumption of pineapple can control body weight and improve hyperlipidemia, AC, CRR including reduced lipid induced-hepatic toxicity in the HCD-fed rats.

### Pineapple consumption decreased HCD-induced oxidative stress in rat’s hearts

**Fig. 4:**
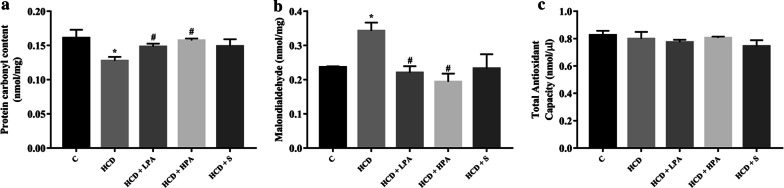
The determination of eight weeks of pineapple consumption on cardiac oxidative stress. Heart tissues were homogenized followed by extraction of protein. **a** Cardiac protein carbonyl content (cPC); **b** malondialdehyde (MDA); **c** total antioxidant capacity (TAC). Each bar presented in mean ± SEM. *p < 0.05 VS the control group, #*p* < 0.05 versus the HCD group, (n = 5–6 per group)

To examine the effect of pineapple consumption on oxidative stress and antioxidant status of the heart, the cardiac protein carbonyl (cPC) content, MDA level and total antioxidant capacities of the HCD model were determined. There was a significance reduction of cPC content in the HCD group (0.13 ± 0.006 nmol/mg versus 0.16 ± 0.012 nmol/mg, *p* < 0.016) but increased the cPC content in both the HCD+LPA and HCD+HPA groups, when compared with the HCD group (0.15 ± 0.004 nmol/mg, 0.016 ± 0.003 nmol/mg versus 0.13 ± 0.006 nmol/mg, *p* < 0.018 and *p* < 0.008, respectively) (Fig. 4a). On the other hand, The MDA level in the HCD group was significantly higher than the control group (0.34 ± 0.024 nmol/mg versus 0.23 ± 0.0026 nmol/mg, *p* < 0.005). After 8-weeks consumption of both the LPA and HPA, the MDA level was significantly lower than the HCD group (0.22 ± 0.019 nmol/mg, 0.019 ± 0.024 nmol/mg versus 0.34 ± 0.024 nmol/mg, *p* < 0.004 and *p* < 0.001, respectively) (Fig. 4b). However, there were no significant differences of the total antioxidant capacity in all the groups (Fig. 4c). These results revealed that daily intake of pineapple can reduce the oxidative modified lipids, but not the proteins, and does not modulate the antioxidant activities in the hearts of the HCD-fed rats.

### Pineapple consumption reduced cardiac inflammation in the HCD fed rats

**Fig. 5:**
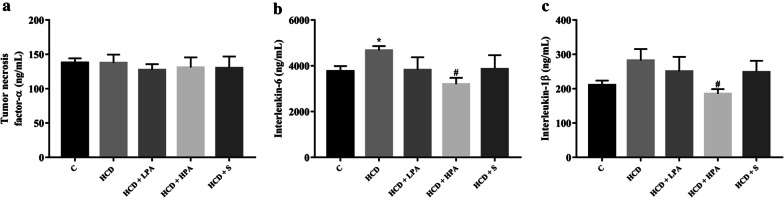
The determination of eight weeks of pineapple consumption on cardiac inflammation. Inflammatory cytokines were analysed by ELISA. **a** Tumor necrosis factor-alpha (TNF-α); **b** interleukin-6 (IL-6); **c** interleukin-1 beta (IL-1β). Each bar presented in mean ± SEM. *p < 0.05 VS the control group, #*p* < 0.05 versus the HCD group, (n = 5–6 per group)

To explore the anti-inflammatory effect of pineapple consumption in HCD-induced cardiac inflammation, heart tissues were harvested to determine the level of the inflammatory cytokines. The results showed no significant difference of the cardiac TNF-α level in all groups (Fig. 5a). The HCD group showed a higher cardiac IL-6 level (4760 ± 192.5 ng/mL versus 3760 ± 227.2 ng/mL, *p* < 0.026) (Fig. 5b), with an increasing trend of the cardiac IL-1β level when compared to the control group (Fig. 5c). After administering HPA for 8-weeks, the cardiac IL-6 and IL-1β levels in the HCD-fed rats were significantly reduced, compared to the HCD group (3197 ± 278.2 ng/mL versus 4,760 ± 192.5 ng/mL, *p* < 0.016 and 185 ± 13.67 ng/mL versus 282.1 ± 33.29 ng/mL, *p* < 0.013) (Fig. 5b, c), while the treatment of simvastatin revealed a tendency to reduce both the cardiac IL-6 and IL-1β levels. These results demonstrated that the consumption of HPA ameliorated HCD-induced cardiac inflammation.

## Discussion

The major findings in the present study demonstrated that pineapple contains potent radical scavenging phenolic and flavonoid compounds. In an animal model, daily intake of pineapple offers significant health benefits for body weight control, anti-dyslipidaemia and reduced hepatic injury in the HCD-induced hypercholesterolemic rats. It was also found that pineapple consumption reduced hypercholesterolemia-induced myocardia lipid peroxidation and inflammation.

A typical feature of hyperlipidaemia is an excessive lipid accumulation in the body, which is prone to promoting obesity [5, 18]. In this study, the HCD group significantly increased BW after two weeks of treatment. Interestingly, both pineapple consumption and simvastatin treatment significantly attenuated the HCD-induced body weight gain without affecting heart to body weight ratio. In comparison to the HCD group, a daily high-dose of pineapple consumption showed a 35 and 40.5% reduction of TC and LDL-C serum, respectively, as well as approximately 46% of simvastatin. The tendency of lipid and BW reduction was similar to the simvastatin treatment and the efficacy of weight control by pineapple consumption was related to the lowering of lipid serum. The daily intake of pineapple did not affect the appetite and food consumption of the rats. Similarly, El-Shazly et al. found that pineapple juice suppressed HFD-induced obesity by decreasing lipid serum, weight gain, and hepatic lipid accumulation through decreased lipogenesis and increased lipolysis at the transcriptional level of lipid metabolism-related genes [18].

It is well-known that dietary fibre plays an essential role in promoting several physiological and metabolic beneficial effects, particularly as a bulking agent, normalising intestinal motility, preventing constipation, and decreasing the intestinal absorption of cholesterol and glucose [27, 28]. The dried pineapple used in this study contains a high dietary fibre content of approximately 9% by weight. It was also recently reported that a high-fibre diet prevented corpulence and decreased TC, TG, LDL-C levels in obesity [29]. In addition, it also provided benefits for the gastrointestinal tract included increased pre-biotic, the shifting of gut microbiota, and enhancing bacterial fermentation in relation to short-chain fatty acids (SCFAs) production [30], which is closely associated with body weight control and the prevention of hyperlipidaemia [30, 31]. However, the effects of pineapple consumption and the role of gut microbiota in hypercholesterolemia requires further investigation. Moreover, evidences suggest that simvastatin reduces the risk of cardiovascular events [32, 33]. Atherogenic indexes of atherogenic and antiatherogenic lipoproteins have emerged as predictive markers for cardiovascular diseases [24]. The atherogenic coefficient and the cardiac risk ratio are powerful indicators of cardiovascular risk; the higher the value, the higher the risk of developing coronary heart disease (CHD) [34]. Daily consumption of pineapple reduced the atherogenic coefficient and cardiac risk ratio in a similar manner to the simvastatin treatment. Therefore, pineapple is an ideal addition to a daily diet, because it can provide an additional lipid-lowering effect which could enable the reduction of medication and serve as a potential functional food for cardiovascular health.

Hypercholesterolemia is associated with elevated cardiac oxidative stress [10]. However, the effect of pineapple consumption on the heart particularly oxidative stress and the inflammation of hypercholesterolemia remains poorly understood. The heart is a highly rated metabolic organ, primarily fuelled by lipids that uptake FAs from the circulation [2, 3]. Critical changes in substrate availability causes cardiac metabolism alterations. Excess circulation of FA levels in hypercholesterolemia creates an imbalance of the lipid uptake to the heart causing lipid accumulation [2]. Therefore, fuel substrates were shifted from glucose to triglycerides and increased fatty acid oxidation (FAO) that induced the overproduction of the reactive oxygen species (ROS), which in turn damaged the biomolecules [2, 3, 35]. Similarly, studies in metabolic syndrome models of Zucker rats and the *db/db* mice demonstrated reduced cardiac glucose oxidation, increased FAO, lipid accumulation, and cardiac dysfunction [2]. We explored the effects of pineapple consumption on cardiac oxidative stress in hypercholesterolemia, while MDA and cPC contents were used as biomarkers for lipid peroxidation and protein oxidation, respectively.

Our data demonstrated that reducing protein oxidation in an HCD-induced hypercholesterolemia model when compared to a normal myocardium. Inversely, pineapple consumption increased the protein carbonylation to a similar level as that of the simvastatin treatment and the control group. However, the elevated protein oxidation level from pineapple consumption which was similar to the control group was questionable.

This could be a possibility for an adaptive response to the HCD intake. The identification of specific modified proteins and their functions particularly in cardiac consequences of pineapple consumption requires further investigation. Cardiac lipid peroxidation increased in the HCD-induced hypercholesterolemia model, while it significantly ameliorated lipid peroxidation after pineapple consumption or simvastatin treatment. Since pineapple contains antioxidant phenolic and flavonoid compounds, its intake could reduce hypercholesterolemia-mediated oxidative stress. Therefore, pineapple consumption is capable of attenuating cardiac oxidative stress on biomolecules indicating that it possesses cardiac antioxidant properties.

Hypercholesterolemia, being overweight and oxidative stress can induce systemic and vital organ inflammation particularly the heart through overexpression of the pro-inflammatory cytokines which are TNF-α, IL-6, and IL-1 beta [35]. This study showed an elevation of cardiac inflammation by increasing IL-6 in the HCD group. This is *the first report* to address the cardioprotective effect of pineapple that attenuates cardiac inflammation in HCD-induced hypercholesterolemia model. IL-6 mediated inflammation is also implicated in cardiovascular diseases [36] which is produced in response to host cell injury, while chronically elevated levels of it could lead to chronic inflammation and fibrotic disorders [36, 37]. The superior anti-inflammatory effect of pineapples can create therapeutic opportunities to reduce IL-6 induced cardiac injury. The polyphenolic compounds found in several fruits, especially pineapples, can inhibit IL-6 mediated inflammation [36, 37].

Regarding the safety of pineapple consumption, the hepatic enzymes and renal function were assessed for adverse effects [38]. Elevated serum levels of AST and ALT from HCD intake suggested that hypercholesterolemia induced hepatic injury. Daily pineapple consumption (in sub-chronic duration) or simvastatin treatment can reduce HCD-induced hepatotoxicity, while renal function remained unaffected in all treated groups. Taking into consideration that daily pineapple consumption has no adverse effects, several studies have reported its beneficial effects for decreasing hepatic lipid accumulation, as well as hepatic injury in HFD and alcohol-induced oxidative stressed models [18, 19]. Pineapple consumption of 100–200 mg/kg in the animal model is equivalent to 37.5–75 g of fresh pineapple for adults weighing 50 kg. The cardioprotective properties of pineapple are affordable and should be incorporated into daily diets.

Overall, daily intake of pineapple provides benefits to health and could be considered as a functional food for cardiovascular condition caused by HCD. The major polyphenolics in pineapples, the molecular mechanisms of antioxidative stress and anti-inflammation in the heart tissue as well as its long-term effects requires further investigation for safe and effective implementation.

## Conclusions

Pineapple possesses antioxidant and lipid-lowering properties, therefore, daily consumption can reduce hypercholesterolemia-induced cardiac lipid peroxidation and pro-inflammation in an in vivo model. This study has demonstrated that pineapple is a potential candidate for cardioprotection against hypercholesterolemia.

## Data Availability

The datasets during and/or analysed during the current study available from the corresponding author on reasonable request.

## Abbreviations

HCD: High-cholesterol diet
AC: Atherogenic coefficient
CRR: Cardiac risk ratio
cPC: Cardiac protein carbonyl
MDA: Malondialdehyde
ASCVD: Atherosclerotic cardiovascular disease
LPA: Low-dose pineapple
HPA: High-dose pineapple
S: Simvastatin
TC: Total cholesterol
HDL-C: High-density lipoprotein cholesterol
LDL-C: Low-density lipoprotein cholesterol
TG: Triglyceride
AST: Aspartate amino-transferase
ALT: Alanine amino-transferase
BUN: Blood urea nitrogen
Cr: Creatinine
TNF-α: Tumor necrosis factor-alpha
IL1-β: Interleukin 1-beta
IL-6: Interleukin 6
TAC: Total antioxidant capacity
SCFAs: Short-chain fatty acids
CHD: Coronary heart disease
FAO: Fatty acid oxidation
